# Emaciation, Congestive Heart Failure, and Systemic Amyloidosis in Severe Recessive Dystrophic Epidermolysis Bullosa: Possible Internal Complications Due to Skin-Derived Inflammatory Cytokines Derived from the Injured Skin

**DOI:** 10.3390/dermatopathology7020007

**Published:** 2020-09-14

**Authors:** Yoshiaki Matsushima, Kento Mizutani, Hiroyuki Goto, Takehisa Nakanishi, Makoto Kondo, Koji Habe, Kenichi Isoda, Hitoshi Mizutani, Keiichi Yamanaka

**Affiliations:** Department of Dermatology, Mie University Graduate School of Medicine, Tsu, Mie 514-8507, Japan; matsushima-y@clin.medic.mie-u.ac.jp (Y.M.); k-mizutani@clin.medic.mie-u.ac.jp (K.M.); onnel25@gmail.com (H.G.); takehisanakanishi@gmail.com (T.N.); pjskt886@yahoo.co.jp (M.K.); habe-k@clin.medic.mie-u.ac.jp (K.H.); isoda@miehifu.jp (K.I.); mzjin@ztv.ne.jp (H.M.)

**Keywords:** recessive dystrophic epidermolysis bullosa, keratinocyte, cytokine, IL-1, emaciation, cardiomegaly, amyloidosis

## Abstract

Inherited epidermolysis bullosa (EB) is a rare genetic skin disorder characterized by epithelial tissue fragility. Recessive dystrophic epidermolysis bullosa (RDEB) is the most severe form, characterized by the presence of blisters, erosion, and ulcer formation, leading to scarring and contraction of the limbs. RDEB is also associated with extra-cutaneous complications, including emaciation, congestive heart failure, and systemic amyloidosis. The main cause of these clinical complications is unknown; however, we hypothesized that they are caused by elevated circulating inflammatory cytokines overproduced by injured keratinocytes. We addressed this phenomenon using keratin-14 driven, caspase-1 overexpressing, transgenic (KCASP1Tg) mice in which injured keratinocytes release high levels of IL-1α and β. KCASP1Tg showed severe spontaneous dermatitis, as well as systemic complications, including aberrant weight loss, cardiovascular disease, and extensive amyloid deposition with organ dysfunction, resembling the complications observed in severe EB. These morbid conditions were partially ameliorated by simultaneous administration of anti-IL-1α and β antibodies. The skin not only constitutes a physical barrier, but also functions as the largest immune organ. We suggest a novel role for IL-1 in the pathogenesis of EB and the use of anti-IL-1 antibodies as a potential therapy for EB complications.

## 1. Introduction

Epidermolysis bullosa (EB) is a rare inherited genetic disease of the skin and mucous membranes. EB is classified into four major subtypes: EB simplex, junctional EB (JEB), dystrophic EB (DEB), and Kindler EB. Recessive DEB (RDEB), formerly called Hallopeau–Siemens-type DEB, is the most severe form of EB [1]. In this subtype, the collagen VII (COL7A1) gene is mutated, and its protein is absent or markedly reduced. Epithelial tissues are fragile, and blisters, erosions, and ulcers are characteristic phenotypes that cause scarring and contraction of the limbs. RDEB is also associated with the risk of skin malignancies, including squamous cell carcinoma (SCC). SCCs occur primarily in RDEB, and cumulative risks rise steeply in severe RDEB, from 7.5% by age 20 to 67.8%, 80.2%, and 90.1% at 35, 45, and 55 years, respectively [2]. Other major extra-cutaneous complications include emaciation, congestive heart failure, and systemic amyloidosis. Emaciation is frequently observed, and congestive heart failure is occasionally detected, even at a young age [3]. Renal amyloidosis causes renal failure and a cumulative risk of death of 12.3% by 35 years of age [4,5,6], but these complications may even be present in milder subtypes of JEB. To date, the main cause of this pathological syndrome in patients with EB remains largely unknown.

## 2. Case Report

**Figure 1:**
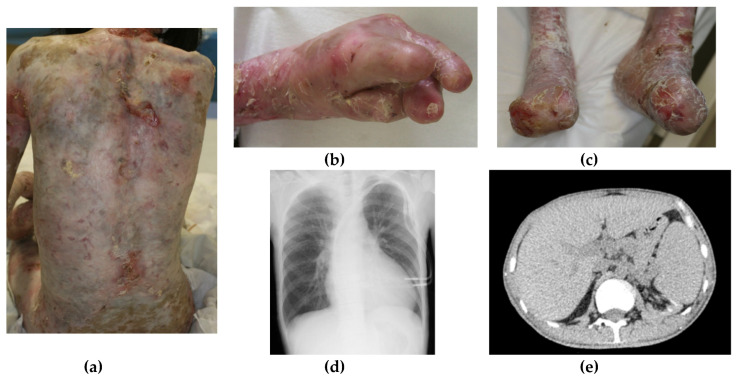
A 37-year-old severe recessive dystrophic epidermolysis bullosa patient is presented. (**a**–**c**) Blister and erosion had been observed beginning soon after his birth, resulting in scarring, contraction, and dactylosymphysis of the limbs; (**d**) a chest radiography revealed cardiomegaly; (**e**) computed tomography showed hepatomegaly and splenomegaly. The diameter of the aorta is narrow in this patient.

Herein, we present the case of a 37-year-old male RDEB patient admitted to our hospital. He had no genetic history, and no genetic tests were performed after admission. The patient experienced chronic blistering and erosion soon after his birth, continuing to admission to the hospital, resulting in scarring, contraction, and dactylosymphysis of the limbs. Repeated transplantation of cultured skin had been performed to treat skin defects. Chest radiography revealed cardiomegaly, and computed tomography showed narrowing of the abdominal aorta (the diameter was 12 mm at the bifurcation of the renal artery (normal—16 mm) and 13 mm at the splenic artery (normal—21 mm)), hepatomegaly and splenomegaly (Figure 1). At 31 years old, squamous cell carcinoma (SCC) was detected at his skin ulcer, and he underwent radical surgery for recurrent SCC nine times. At 32 years old, there was marked deterioration of renal function and congestive heart failure. A renal biopsy was not performed because of the high risk to the patient, but a nephrologist diagnosed him with renal amyloidosis. His serum total protein level was 7.5 g/dL (normal 6.5–8.0 g/dL). The serum albumin level was 2.5 g/dL (normal 3.5–5.0 g/dL). The serum aspartate transaminase (AST) level was 12 U/L (normal <40 U/L). The serum alanine transaminase (ALT) level was 10 U/L (normal <35 U/L). The serum lactate dehydrogenase level was 110 IU/L (normal 100–230 IU/L). The serum C-reactive protein (CRP) level was 13.47 mg/dL (normal <0.30 mg/dL). The serum amyloid A (SAA) level was 1250 µg/mL (normal 0–8 µg/mL). The serum immunoglobulin G level was 2040 mg/dL (normal 800–1800 mg/dL). The serum rheumatoid factor level was 2 U/mL (normal <20 U/mL). Serum IL-1α and IL-1β levels were measured, but IL-1α levels were below the detection limit, and IL-1β was not significantly elevated, as compared with the normal control, which was partially due to the sensitivity of the system. The serum IL-18 level was 512.1 pg/mL (healthy control: 90.8 pg/mL), and the IL-17A level was more than five-time higher compared to healthy controls (data not shown). The serum creatinine level was 4.65 mg/dL (normal 0.60–1.40 mg/dL). The 24-h urinary protein level was 2.24 g/day (normal 0.02–0.06 g/day). Two years after the deterioration of his renal function, he started treatment with artificial hemodialysis. At 37 years old, he died due to the progression of emaciation. This study was approved by the ethics committee of Mie University (#2684).

## 3. Discussion

We supposed that the organ involvement observed in EB patients is mainly associated with inflammatory cytokines derived from injured skin. Epidermal keratinocytes store active IL-1α and the precursor of IL-1β, which are key cytokines for initiating the inflammatory response and activating the cytokine cascade [7,8]. IL-1α is processed and secreted in inflamed skin lesions. IL-1β is stored as an inactive precursor and can be activated by specific enzymes before being secreted. In EB, progression of the pathological skin phenotype is accelerated by inflammation and minor trauma, as previously demonstrated [8,9]. IL-1β signaling is constitutively activated in EB keratinocytes [10], and serum IL-1α and β levels are several-fold increased, even in the mild form of SEB [11]. Other inflammatory cytokines, including IL-6 and IL-17A, may be involved in the acceleration of the inflammatory cascade. IL-6 is a known downstream target of IL-1β. After IL-6 is synthesized in a local lesion, it circulates to the liver through the bloodstream, followed by the rapid induction of an extensive range of acute phase proteins, such as CRP, SAA, and fibrinogen. IL-6 is involved in the regulation of T cell differentiation between regulatory T (Treg) and T helper 17 (Th17) cells. IL-6 triggers the differentiation of Th17 cells, together with TGF-β, by enhancing RORγt expression and suppresses the generation of Treg cells via STAT3 activation. Th17 cells secrete several pro-inflammatory cytokines, such as IL-17A, and initiate various inflammatory responses. Furthermore, diminished Tregs worsen the situation. IL-6 functions upstream of IL-17A and acts as a critical downstream target of IL-17A. In vivo expression of IL-17A increases serum IL-6 concentration. IL-17A and IL-6 also synergistically induce the expression of various NF-κB target genes.

**Figure 2:**
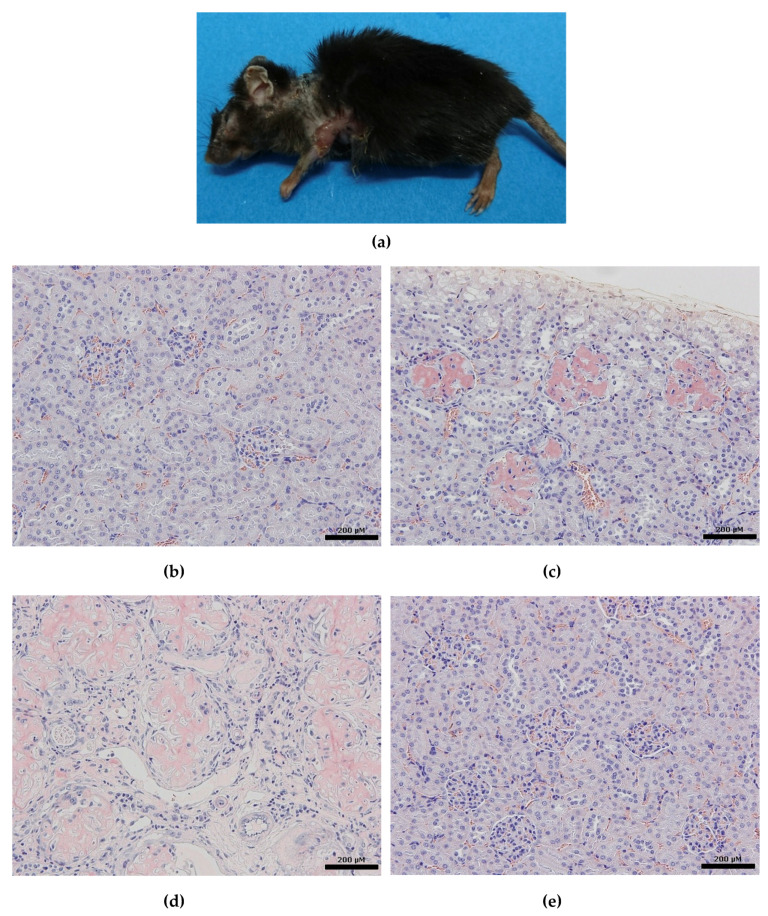
(**a**) Severe erosive dermatitis in a keratin-14 driven caspase-1 overexpressing transgenic (KCASP1Tg) mouse gradually spreads across the entire face and trunk, covering approximately 15% of the body surface, resulting in scarring and contraction at 5 months old; (**b**) no changes in the kidney and glomerulus of an 8-week-old KCASP1Tg mouse (Congo red staining (CR), bar 200 μM); (**c**) in a 16 week old KCASP1Tg mouse, amyloid is deposited in the glomerulus (CR, bar 200 μM); (**d**) a 24-week-old KCASP1Tg mouse has increased amyloid deposits in the glomerulus and stroma (CR, bar 200 μM); (**e**) in wild-type mice, amyloid deposition was not observed, even at 24 weeks old (CR, bar 200 μM).

Chronic inflammation causes aberrant remodeling of vascular and fatty tissues, leading to atherosclerosis and obesity/lipodystrophy [12]. Inflammatory cytokines are believed to primarily affect the surrounding cells at sites of tissue injury. In addition to their primary role as a local mediator, excessive expression of inflammatory cytokines spills over into the systemic circulation and affects remote organs. Sustained skin disruption in EB patients can lead to aberrant cytokine secretion that may potentially cause vascular and visceral pathologies. We addressed this problem by using our original inflammatory skin model mice: a keratin 14 overexpressing caspase-1 transgenic (KCASP1Tg) mouse in which keratinocytes release high levels of IL-1 and IL-18 (serum IL-1α level—32–40 pg/mL, serum IL-1β level—40–45 pg/mL, and serum IL-18 level—8–11 ng/mL) [12]. Caspase-1 cleaves the endogenous precursors of IL-1β and IL-18 and converts them to their active forms. Although both IL-1β and IL-18 were elevated in KCASP1Tg mice, IL-1β was considered a more critical cytokine in the progression of emaciation, compared to IL-18 [12,13]. IL-18 is a silent cytokine that becomes active together with IL-12 to induce a Th1 immune response. In fact, although keratin 14 overexpressing IL-18 transgenic mice produce IL-18 from early development, no clinical phenotype occurs. However, once eruptions occur, skin lesions produce and release IL-1β and other inflammatory cytokines, which then induce emaciation, aortic sclerosis, and systemic amyloidosis [13,14,15]. The KCASP1Tg mouse developed erosive dermatitis from the age of 8 weeks, which gradually spread across the entire face and trunk, covering approximately 15% of the body surface, and resulting in scarring and contraction when the mouse was 5 months old (Figure 2a). Notably, the KCASP1Tg mice gradually became emaciated, accompanied by a loss of visceral fat as the dermatitis spread because of adipocyte atrophy and degeneration due to circulating cytokines [13]. As with EB, KCASP1Tg mice also showed arteriosclerosis obliterans (ASO) and cardiomegaly [13]. The pathogenesis of ASO has not been elucidated completely, but we speculated that long-lasting, low-grade hypercytokinemia directly damages the endothelial cells and vascular components. Extensive amyloid deposition with organ dysfunction was also detected, resembling the complications observed in severe EB. We investigated whether these symptoms occurring in inflammatory mice could be improved by anti-IL-1 antibody treatment. Ten micrograms of anti-IL-1α, β, and both α plus β neutralizing antibodies (BioLegend, San Diego, CA, USA) were injected intraperitoneally into KCASP1Tg mice once a week from 1 month to 6 months of age. These pathologies were ameliorated by simultaneous treatment with anti-IL-1α and anti-IL-1β antibodies [12]. Like the present case, renal amyloidosis also occurs in KCASP1Tg mice. It is no different from wild mice until dermatitis occurs, but as dermatitis persists, amyloid is gradually deposited in the glomerulus and further into the interstitium, and the renal structure is destroyed (Figure 2b–e). This is probably due to increased SAA produced in the liver and immune globulin deposition [16]. In a study by Esposito et al., most cytokines with inflammatory activity (i.e., IL-1β, IL-2 IL-6, TNF-β, and INF-γ) were significantly higher in EB patients than in healthy controls. They were higher in RDEB patients than in other EB patients [17], as well. Therefore, the same phenomenon detected in our inflammation model mice may occur in these patients.

## 4. Conclusions

Long-lasting and severe disruption of the skin is observed with EB. Epidermal keratinocytes act as pivotal immune sentinels; after stimulation with exogenous factors, keratinocytes release cytokines. We demonstrated in mice with long-lasting, low-grade hypercytokinemia including IL-1 and other inflammatory cytokines derived from severe skin damage causes serious complications in multiple organs; these findings are similar to those observed in the most severe form of EB. In our mouse model, this morbid syndrome was partially ameliorated by simultaneous treatment with anti-IL-1α and β antibodies, or in mice generated by crossing KCASP1Tg mice with IL-1α/β double knock-out mice [13,18], suggesting the potential therapeutic value of anti-cytokine therapy for these complications associated with EB.
